# Structure of the Tandem Fibronectin Type 3 Domains of Neural Cell Adhesion Molecule

**DOI:** 10.1016/j.jmb.2008.01.030

**Published:** 2008-03-21

**Authors:** Federico Carafoli, Jane L. Saffell, Erhard Hohenester

**Affiliations:** Department of Life Sciences, Biophysics Section, Blackett Laboratory, Imperial College London, London SW7 2AZ, UK

**Keywords:** CAM, cell adhesion molecule, NCAM, neural cell adhesion molecule, Ig, immunoglobulin, FN3, fibronectin type 3, FGFR, fibroblast growth factor receptor, SPR, surface plasmon resonance, RU, resonance units, PBS, phosphate-buffered saline, TBS, Tris-buffered saline, cell adhesion, domain linker, crystal packing, protein interaction, X-ray crystallography

## Abstract

Activation of the fibroblast growth factor receptor (FGFR) by neural cell adhesion molecule (NCAM) is essential for NCAM-mediated neurite outgrowth. Previous peptide studies have identified two regions in the fibronectin type 3 (FN3)-like domains of NCAM as being important for these activities. Here we report the crystal structure of the NCAM FN3 domain tandem, which reveals an acutely bent domain arrangement. Mutation of a non-conserved surface residue (M610R) led to a second crystal form showing a substantially different conformation. Thus, the FN3 domain linker is highly flexible, suggesting that it corresponds to the hinge seen in electron micrographs of NCAM. The two putative FGFR1-binding segments, one in each NCAM FN3 domain, are situated close to the domain interface. They form a contiguous patch in the more severely bent conformation but become separated upon straightening of the FN3 tandem, suggesting that conformational changes within NCAM may modulate FGFR1 activation. Surface plasmon resonance experiments demonstrated only a very weak interaction between the NCAM FN3 tandem and soluble FGFR1 proteins expressed in mammalian cells (dissociation constant > 100 μM). Thus, the NCAM–FGFR1 interaction at the cell surface is likely to depend upon avidity effects due to receptor clustering.

## Introduction

The neural cell adhesion molecule (NCAM) is the prototype and founding member of the immunoglobulin (Ig) superfamily cell adhesion molecules (CAMs).^1–4^ NCAM is present on the cell surface of neurons, astrocytes and oligodendrocytes, where it mediates homophilic and heterophilic cell adhesion. NCAM is involved in neuronal migration, axon growth and guidance, as well as in synaptic plasticity associated with learning and memory.^5–7^ Alternative splicing of the *NCAM1* gene results in isoforms of three size classes that differ in their membrane attachment and cytosolic regions but have in common an extracellular domain consisting of five Igs and two fibronectin type 3 (FN3) domains.^8^ The two larger isoforms have a transmembrane helix and cytosolic domains of different sizes, while the smallest isoform has a glycophosphatidylinositol membrane anchor. Variable use of alternative exons in the extracellular domain results in small insertions into Ig4 or between the FN3 domains.^9–12^ NCAM function is further regulated by an unusual posttranslational modification, namely, the addition of polysialic acid to Ig5.^13^

The molecular basis of homophilic adhesion by NCAM has been a subject of intense study, and the results from biochemical and biophysical studies have not always been consistent.^14,15^ A crystal structure of NCAM Ig1–Ig3 has led to a zipper model of adhesion, which postulates both *cis* and *trans* interactions by the Ig1–Ig3 region of NCAM.^16^ While a conclusive picture has yet to emerge, it appears that there may be multiple modes of homophilic interaction.^17^

**Fig. 1:**
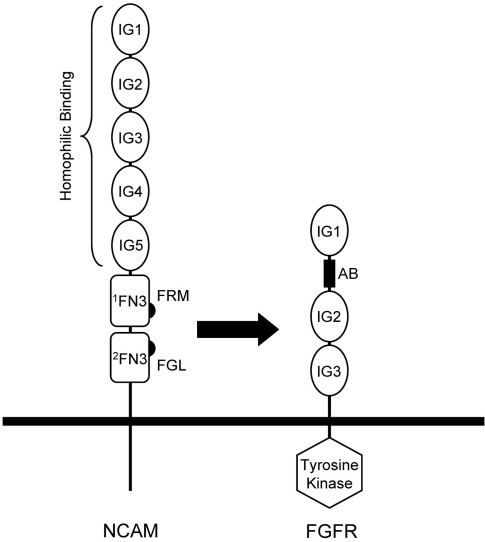
Schematic drawing of NCAM and FGFR at the cell surface. The cell membrane is represented by a thick horizontal line. Ig and FN3 domains are represented by ovals and rectangles, respectively. The cytosolic tyrosine kinase domain of FGFR is represented by a hexagon; the acid box (AB; see the text) is represented by a filled black rectangle. The arrow indicates the interaction of two NCAM regions (FRM and FGL; see the text) with FGFR.

NCAM is also engaged in heterophilic interactions. There is now convincing evidence that NCAM-mediated neurite outgrowth, as well as tumour development and progression, critically involves the activation of fibroblast growth factor receptor 1 (FGFR1), through a *cis* interaction of NCAM and FGFR1.^18–22^ The four FGFRs and their 23 growth factor ligands control a variety of cellular processes, including development, angiogenesis, hematopoiesis and tumourigenesis.^23–26^ Alternative splicing of the four FGFR genes results in at least 48 receptor isoforms that vary in their ligand binding profiles and kinase domains. The longest FGFR1 ectodomain variant consists of three Ig domains, D1–D3, with a stretch of acidic amino acids (the “acid box”) inserted into the D1–D2 linker; shorter forms lack D1 and the acid box. The NCAM–FGFR1 interaction was originally proposed based on indirect biological evidence,^27^ but biochemical evidence has been obtained to suggest that the interaction is mediated by direct binding of the FN3 domains of NCAM to FGFRs (Fig. 1).^20,28,29^ We and others identified NCAM-derived peptides capable of stimulating FGFR1 signalling and inducing neurite outgrowth. One peptide (FRM peptide) is derived from the first FN3 domain of NCAM (^1^FN3),^30^ while another (FGL peptide) is derived from the second FN3 domain (^2^FN3).^28^ The structures of the ^1^FN3 and ^2^FN3 domains in isolation have been determined,^28,31^ but how the two domains cooperate in FGFR1 activation is unclear. In this study, we determined the crystal structure of the NCAM FN3 tandem (^1^FN3–^2^FN3) in two crystal forms. We report that ^1^FN3–^2^FN3 assumes a bent conformation in both forms, with evidence of substantial flexibility of the domain linker. In direct binding experiments with fully glycosylated proteins of mammalian origin, we observed only a very weak interaction (dissociation constant > 100 μM) of NCAM ^1^FN3–^2^FN3 with two FGFR1 ectodomain constructs. We conclude that the NCAM–FGFR1 interaction at the cell surface may be transient or stabilised by avidity effects resulting from receptor clustering and that conformational changes within NCAM may have a profound role in FGFR1 activation.

## Results

### Crystal structure of the NCAM FN3 tandem

**Fig. 2:**
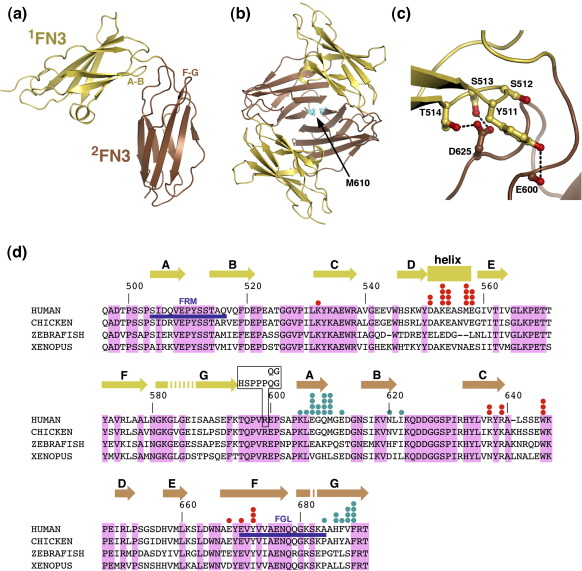
Structure of the NCAM FN3 tandem. (a) Cartoon drawing of the wild-type ^1^FN3–^2^FN3 structure. Two loops implicated in FGFR1 binding (see the text) are labelled. (b) Cartoon drawing of the ^1^FN3–^2^FN3 dimer viewed along the 2-fold non-crystallographic symmetry axis. The ^1^FN3 and ^2^FN3 domains are shown in yellow and brown, respectively. The side chain of M610 in the dimer interface (see the text) is shown as a ball-and-stick model and is labelled. (c) Close-up view of the domain interface in wild-type ^1^FN3–^2^FN3: ^1^FN3 is shown in yellow; ^2^FN3, in brown. Selected residues are shown as ball-and-stick models. Hydrogen bonds are indicated by dashed lines. (d) Sequence alignment of the FN3 tandem of selected vertebrate NCAMs. Conserved residues are shaded pink. The numbering scheme and secondary structure elements of human NCAM are indicated above the alignment. The alternative splice inserts in the ^1^FN3–^2^FN3 linker (see the text) are indicated by black boxing. Two sequences implicated in FGFR1 binding (see the text) are underlined in blue. Residues involved in forming the dimer shown in (b) are indicated by filled circles, with the number of circles being proportional to the accessible surface area buried in the dimer: red circles indicate dimer contact between the α-helix of ^1^FN3 and the GFCD sheet of ^2^FN3; cyan circles, dimer contact between β-strands A and G of ^2^FN3.

To obtain insight into the relative orientation of the two FN3 domains of NCAM, we determined the crystal structure of ^1^FN3–^2^FN3 at 2.3-Å resolution (Table 1). Both ^1^FN3 and ^2^FN3 adopt the typical β-sandwich fold of all FN3 domains consisting of seven strands arranged in two antiparallel sheets (ABE and GFCD) (Fig. 2a and b). Preceding strand A in both domains are short proline-rich segments that are integrated into the FN3 fold, with the proline tetrahydropyrrole rings pointing into the hydrophobic core (Pro500 and Pro503 in ^1^FN3; Pro601 and Pro604 in ^2^FN3). A similar feature has been observed in other FN3 domains (e.g., in gp130^33^ and titin^34^). ^1^FN3 contains an unusual α-helix situated between strands D and E, as reported previously.^31^ ^1^FN3 in our FN3 tandem structure matches the crystal structure of ^1^FN3 in isolation,^31^ with an r.m.s.d. of 0.50 Å for 100 C^α^ atoms. ^2^FN3 in our FN3 tandem matches the solution structure of ^2^FN3 in isolation,^28^ with an r.m.s.d. of 1.3 Å for 92 C^α^ atoms (the main differences are concentrated in the B–C and C–D loops).

The relative orientation of the two FN3 domains in the ^1^FN3–^2^FN3 tandem is characterised by an unusually bent conformation with an interdomain angle of ∼ 80° (calculated between the long axes of the two FN3 domains). The interface between ^1^FN3 and ^2^FN3 buries 630 Å^2^ of solvent-accessible surface (calculated with the CCP4 program AREAIMOL), which is in the typical range for rigid FN3 (and Ig) domain interfaces. The interface is dominated by polar interactions, between the A–B loop of ^1^FN3 on the one hand and the domain linker and B–C loop of ^2^FN3 on the other (Fig. 2c) The key interface residues (Tyr511, Ser513, Thr514, Pro601 and Asp625) are strictly conserved in all vertebrate NCAM sequences (Fig. 2d), suggesting that the bent conformation may be physiologically relevant. However, residues 511–514 have also been implicated in FGFR1 binding,^30^ and the linker region could assume a very different structure when NCAM is bound to FGFR1 (see below).

The conformation of multidomain proteins often is influenced by the crystal lattice. In this regard, we noted that the asymmetric unit of our crystals contains two ^1^FN3–^2^FN3 molecules arranged in a tightly interlocked dimer (Fig. 2b). There are two main contacts responsible for dimer formation: the α-helix of ^1^FN3 packs against the GFCD sheet of ^2^FN3 of the other molecule, and the ^2^FN3 domains of the two molecules interact via their A and G strands. Altogether, these contacts bury as much as 3040Å^2^ of solvent-accessible surface. We do not think that the dimer observed in our crystals is physiologically relevant, as dimer interface residues are only poorly conserved (Fig. 2d) and NCAM dimers were never observed in electron microscopy studies.^35,36^ Because we were concerned that the tight association of ^1^FN3–^2^FN3 molecules in the dimer may have forced the unusually bent interdomain conformation, we sought to disrupt the dimer by mutagenesis and crystallise a mutant ^1^FN3–^2^FN3 protein in a different crystal form.

### Structure of the M610R mutant

**Fig. 3:**
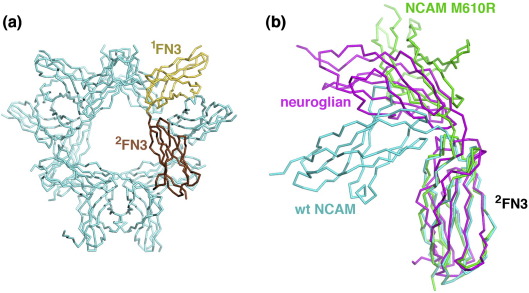
Structure of M610R mutant. (a) C^α^ trace of the structure of the NCAM ^1^FN3–^2^FN3 M610R mutant, viewed along the 3-fold non-crystallographic symmetry axis. One molecule is highlighted in yellow (^1^FN3 domain) and brown (^2^FN3 domains). (b) Superposition of the FN3 pairs of wild-type NCAM (cyan), NCAM M610R mutant (green) and neuroglian (magenta).^37^ The structures were superimposed on the conserved β-strands of the second FN3 domain. wt indicates wild type.

We expressed three point mutants of NCAM ^1^FN3–^2^FN3 (M610R, Y672E and R690E), all of which should be incompatible with the dimer structure seen in crystals of the wild-type protein. Importantly, all three mutations target surface residues and are unlikely to have an effect on NCAM folding. Whether the mutations are functionally neutral could not be determined due to a lack of suitable assays. When examined by size-exclusion chromatography, all three mutants eluted as a mixture of monomers and dimers, similar to the wild-type protein (data not shown). We obtained crystals of the M610R mutant and determined its structure at 2.7-Å resolution by molecular replacement (Fig. 3a). The asymmetric unit of the crystals contains a hexamer of NCAM ^1^FN3–^2^FN3 M610R. The hexamer can be regarded as a trimer of dimers, with the dimers having a completely different mode of association compared with the wild-type structure (the individual domains are very similar, as expected, with r.m.s.d. values of 0.93 and 1.1 Å for ^1^FN3 and ^2^FN3, respectively). The relative orientation of the two FN3 domains in the M610R mutant is less severely bent than in the wild-type structure but still far from fully extended (interdomain angle ∼ 120°; Fig. 3b). The FN3 pair opens up in the M610R mutant, and ^1^FN3 is additionally twisted about its long axis. The combined hinge opening and ^1^FN3 twisting amounts to a pure rotation of 73° for ^1^FN3 when the structures are superimposed on their ^2^FN3 domains. There is no domain interface to speak of in the mutant (240 Å^2^ buried), and the conformation appears to be stabilised entirely by the crystal lattice. Thus, crystal lattice forces can have a profound influence on the conformation of the NCAM FN3 tandem, suggesting that the ^1^FN3–^2^FN3 linker may act as a flexible hinge in native NCAM.

We compared the NCAM FN3 tandem with other FN3 tandems of known structure. Tandems from the extracellular matrix proteins fibronectin and tenascin generally assume an extended conformation.^38–40^ In contrast, many cytokine and hormone receptors (e.g., gp130)^33^ feature bent FN3 pairs that are superficially similar to the NCAM structures reported here (data not shown). Of particular interest is the structure of the FN3 pair of neuroglian, a *Drosophila* CAM.^37^ The neuroglian tandem, which has an extensive domain interface that incorporates a bound sodium ion, adopts a conformation that is intermediate between the two conformations we report for the NCAM tandem. Thus, a similar bend in the FN3 linker(s) may be a general feature of other animal CAMs containing FN3 domains in their membrane-proximal region.

### Location of putative FGFR1 binding site

**Fig. 4:**
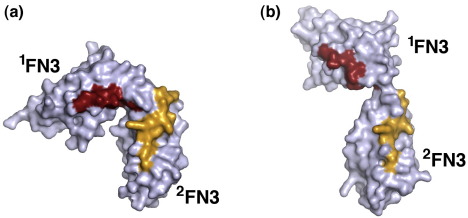
Location of putative FGFR1 binding site. Shown are surface representations of (a) wild-type NCAM ^1^FN3–^2^FN3 and (b) its M610R mutant. The FRM and FGL sequences (see the text) are shown in red and yellow, respectively.

Previous studies have implicated two NCAM ^1^FN3–^2^FN3 regions in FGFR1 binding. Kiselyov *et al.* identified a bioactive peptide from the F–G loop of ^2^FN3 (FGL peptide),^28^ and we identified a bioactive peptide from the A–B loop of ^1^FN3 (FRM peptide).^30^ In agreement with our earlier prediction,^30^ the FRM and FGL loops are indeed located in close proximity on the same face of the wild-type NCAM molecule (Fig. 4a). However, in the M610R mutant, the opening up of the two domains combined with rotation of ^1^FN3 places the FRM and FGL loops much farther apart. Thus, conformational changes at the NCAM ^1^FN3–^2^FN3 hinge may modulate the interaction of NCAM with FGFR1.

### Surface plasmon resonance analysis of the NCAM–FGFR1 interaction

**Fig. 5:**
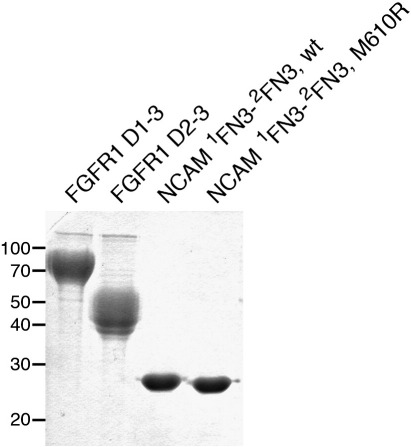
SDS-PAGE analysis of recombinant NCAM and FGFR1 proteins. Coomassie blue-stained gel of His-tagged soluble proteins expressed in 293-EBNA cells. The positions of molecular mass standards (in kilodaltons) are indicated on the left. The FGFR1 proteins are modified by extensive glycosylation; the NCAM proteins are not glycosylated.

**Fig. 6:**
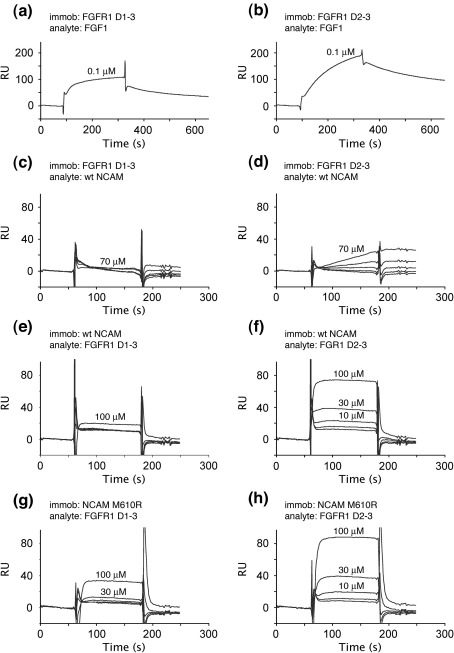
SPR analysis of the NCAM–FGFR1 interaction. Shown are raw sensorgrams obtained on a Biacore 3000 instrument. Selected curves are labelled with the respective analyte concentration. (a) Binding of 0.1 μM FGF1 to immobilised FGFR1 D1–D3. (b) Binding of 0.1 μM FGF1 to immobilised FGFR1 D2–D3. (c) Binding of 4.4, 8.8, 17.5, 35 and 70 μM NCAM ^1^FN3–^2^FN3 to immobilised FGFR1 D1–D3. (d) Binding of 4.4, 8.8, 17.5, 35 and 70 μM NCAM ^1^FN3–^2^FN3 to immobilised FGFR1 D2–D3. (e) Binding of 1, 3, 10, 30 and 100 μM FGFR1 D1–D3 to immobilised wild-type NCAM ^1^FN3–^2^FN3. (f) Binding of 1, 3, 10, 30 and 100 μM FGFR1 D2–D3 to immobilised wild-type NCAM ^1^FN3–^2^FN3. (g) Binding of 1, 3, 10, 30 and 100 μM FGFR1 D1–D3 to immobilised NCAM ^1^FN3–^2^FN3 M610R. (h) Binding of 1, 3, 10, 30 and 100 μM FGFR1 D2–D3 to immobilised NCAM ^1^FN3–^2^FN3 M610R.

We wanted to map the FGFR1 binding site on the NCAM FN3 tandem by structure-based mutagenesis and first sought to establish a suitable binding assay. A solid-phase assay with immobilised NCAM and Fc-tagged FGFR1 proteins did not show any appreciable interaction (data not shown). We therefore used surface plasmon resonance (SPR) to analyse the binding of NCAM ^1^FN3–^2^FN3 to two FGFR1 ectodomain constructs. The FGFR1 D1–D3 construct used (residues 22–364) spans essentially the full ectodomain and contains the acid box situated between domains D1 and D2. The FGFR1 D2–D3 construct used (residues 151–364) lacks D1 and the acid box but retains the binding site for FGFs; this construct is similar to the construct previously used by Kiselyov *et al.* in SPR studies.^28^ Both soluble FGFR1 proteins were produced by the 293-EBNA cells in good yields. Due to the presence of multiple N-linked glycosylation sites in FGFR1 (see below), the purified recombinant proteins migrate as diffuse bands of higher-than-calculated molecular mass on SDS-PAGE (Fig. 5). In a first set of experiments, the two FGFR1 constructs were immobilised on a CM5 sensor chip [8000 resonance units (RU) of D1–D3 and 3850 RU of D2–D3]. Recombinant FGF1 injected at a concentration of 100 nM produced sensorgrams characteristic of a high-affinity interaction, confirming that the immobilised proteins are functional (Fig. 6a and b). In contrast, wild-type NCAM ^1^FN3–^2^FN3 up to a concentration of 70 μM did not produce a signal on the FGFR1 D1–D3 surface and showed only very weak binding to FGFR D2–D3 (Fig. 6c and d). In a second set of experiments, the order of proteins was reversed. NCAM ^1^FN3–^2^FN3 proteins were immobilised on a CM4 sensor chip (1800 RU of wild-type protein and 1900 RU of M610R mutant), and the two soluble FGFR1 constructs were used as analytes up to a concentration of 100 μM. We again observed only weak interactions for all pairings (Fig. 6e–h). Wild-type and M610R NCAM ^1^FN3–^2^FN3 behaved almost identically in these experiments, and, as before, it appeared that the affinity of NCAM for FGFR1 D2–D3 was higher than that for FGFR1 D1–D3. The fast association and dissociation steps in the sensorgrams prevented the fitting of kinetic constants. We used the plateau values at equilibrium to estimate a dissociation constant of > 100 μM for the interaction of NCAM ^1^FN3–^2^FN3 with FGFR1 D2–D3 (not shown), but we emphasise that this value is very approximate given the weak resonance signals obtained. In view of the weakness of the NCAM–FGFR1 interaction in our assay, we were unable to pursue our initial plans of mapping the binding site(s) by mutagenesis.

## Discussion

### NCAM ectodomain structure

The current view of the NCAM ectodomain structure is based on early studies by rotary shadowing electron microscopy of tissue-derived NCAM.^35^ Electron microscopy visualised the NCAM ectodomain as ∼ 28-nm rods bent at a flexible hinge located ∼ 10 nm from the C-terminus; the angle between the two arms varied from 50° to 140° (average = 100°). The hinge was attributed to the proline-rich linker between Ig5 and the first FN3 domain, and the long and short arms were described as rigid domain tandems (Ig1–Ig5 and ^1^FN3–^2^FN3, respectively) in extended conformations.^35^ A crystal structure of NCAM Ig1–Ig3 indeed showed a largely extended structure.^16^ In sharp contrast, the present crystal structure analysis of the NCAM ^1^FN3–^2^FN3 domain pair has revealed a prominent bend between the two domains in two independent crystal forms (Fig. 3b). This finding is difficult to reconcile with the uniformly straight appearance of the short arm in the electron micrographs and suggests that the hinge point may actually lie between ^1^FN3 and ^2^FN3. In this respect, we note that the Ig5–^1^FN3 linker is actually rather short and may well be rigid (in the Ig5–^1^FN3 linker sequence, ILVQADTPSSP, the isoleucine and valine residues are predicted to contribute to the hydrophobic core of Ig5 and the first proline is already part of the ^1^FN3 fold). If the hinge is instead situated in the ^1^FN3–^2^FN3 linker, the juxtamembrane domain of NCAM must have contributed to the short arm seen in electron micrographs, as a single FN3 domain would only account for half of the short arm length. The serine/threonine-rich juxtamembrane domain of NCAM (sequence TSAQPTAIPANGSPTSGLSTGA) is predicted to be extensively modified by O-linked glycosylation (NetOGlyc 3.1 server†) and could easily assume the extended and rigid conformation required to span the remaining ∼ 5 nm.^41^ Further structural analysis, in particular, of the Ig5–^1^FN3 pair, is required to conclusively pinpoint the site of articulation within the NCAM ectodomain.

### NCAM splice variants

Interestingly, the flexible hinge linking the two FN3 domains of NCAM ^1^FN3–^2^FN3 is known to be modified by alternative splicing. Our structure is of the shortest isoform (linker sequence TQPVREPSAP), whereas the underlined arginine residue is replaced by QG, HSPPPQG or even longer sequences in other variants.^9,10,12^ The HSPPPQG insertion has been suggested as a potential hinge region.^1^ The biological relevance of these splicing events is not well understood, but *in vitro* experiments have demonstrated that the NCAM isoforms differ in their capacity to support cell adhesion and spreading,^42,43^ as well as myoblast fusion.^44^ We prepared NCAM ^1^FN3–^2^FN3 proteins with QG or HSPPPQG inserts in the domain linker, but, unfortunately, these proteins were very prone to aggregation in physiological buffers and could not be used for SPR or structural analysis (data not shown).

### Interaction of NCAM with FGF

Hinge bending and alternative splicing at the ^1^FN3–^2^FN3 junction could affect NCAM function by modulating either its homophilic binding properties or its heterophilic interactions with other proteins. There is currently no evidence for the former scenario. In contrast, the functional interaction of NCAM with FGFR1 in cis (i.e., at the same cell membrane) is well established^1,22,30^ and would appear to be an attractive candidate for regulation by alternative splicing. Peptides from two regions of the NCAM ^1^FN3–^2^FN3 tandem, one in each domain, have been shown to modulate FGFR1-dependent neurite outgrowth.^28,30^ Intriguingly, the corresponding loop regions are in close proximity in the acutely bent conformation observed in crystals of the wild-type protein, suggesting that they are part of a larger FGFR1 binding site extending over the domain junction. In crystals of the M610R mutant, the two putative FGFR1-binding loops are farther apart and no longer on the same face of the ^1^FN3–^2^FN3 structure (Fig. 4). Thus, changes in the ^1^FN3–^2^FN3 conformation, either by biomechanical forces resulting from cell–cell contact or by alternative splicing, could have a profound effect on the NCAM–FGFR1 interaction. Other NCAM activities that might be affected by alternative splicing of the ^1^FN3–^2^FN3 linker include interactions with polysialyltransferases^31^ and prion protein,^45^ which both bind to the FN3 domains.

Previous biochemical studies reported a dissociation constant of ∼ 10 μM for the interaction between NCAM ^1^FN3–^2^FN3 and FGFR1 D2–D3.^28,29^ Another study, using cell-based assays, concluded that the acid box in the FGFR1 D1–D2 linker was essential for the NCAM–FGFR1 interaction.^20^ We wanted to use SPR binding experiments to identify NCAM residues involved in FGFR1 binding and test the effect of splice inserts in the ^1^FN3–^2^FN3 linker. Unfortunately, using our recombinant proteins expressed in mammalian cells (Fig. 5), we were unable to detect substantial binding between soluble FGFR1 and NCAM proteins, regardless of whether FGFR1 or NCAM was immobilised on the sensor chip. A very weak interaction (estimated dissociation constant > 100 μM) was evident between FGFR1 D2–D3 and NCAM ^1^FN3–^2^FN3, but we observed no NCAM binding to the FGFR1 D1–D3 construct containing the acid box (Fig. 6). The most likely explanation for the discrepancy between our findings and those of Christensen *et al.*^29^ is the difference in glycosylation of the FGFR1 proteins used. Christensen *et al.* expressed FGFR1 D2–D3 in insect cells, which produce N-linked oligosaccharides of the high-mannose type, whereas our expression system (human embryonic kidney cells) produces complex-type oligosaccharides, which more closely resemble the glycan present on mammalian FGFRs. Human and rodent FGFR1 proteins are highly glycosylated, and the glycan is known to influence ligand binding.^46^ We think that there may be electrostatic repulsion between the acidic NCAM ^1^FN3–^2^FN3 protein (isoelectric point 5.0) and the terminal sialic acids present on FGFR1 expressed in human cells. The very weak interaction we observed between soluble NCAM and FGFR1 proteins does not preclude a critical role of this interaction at the cell surface, where avidity effects due to receptor clustering may be substantial. An attractive hypothesis is that the functional state of NCAM (i.e., whether it is engaged in a homophilic contact or not) is linked to FGFR1 binding and activation. It is tempting to speculate that the flexible ^1^FN3–^2^FN3 linkage revealed by our structural analysis could provide the molecular means for such a regulatory mechanism.

## Experimental Procedures

### Expression vectors

NCAM constructs were made by PCR amplification from a bacterial expression vector coding for the FN3 pair of human NCAM. Our NCAM numbering scheme corresponds to SwissProt entry P13591 up to residue 598 but differs by − 1 from P13591 for all subsequent residues due to the replacement of Gln599–Gly600 by Arg, a naturally occurring splice variant in the brain and muscle.^12^ The M610R mutation in NCAM was introduced by strand-overlap-extension PCR. FGFR1 constructs were made by PCR amplification from a complete cDNA clone of human FGFR1 (IIIc isoform; our numbering scheme corresponds to SwissProt entry P11362). The PCR products were cloned into a modified pCEP-Pu vector^47^ coding for proteins with a C-terminal His_6_ tag. The insert sequences of all expression vectors were verified by DNA sequencing. The domain boundaries of the constructs are as follows: NCAM ^1^FN3–^2^FN3, QADTP…VFRTS (496–692); FGFR1 D1–D3, RPSPT…EALEE (22–364); and FGFR1 D2–D3, VAPYW…EALEE (151–364). Vector-derived APLA and AAAHHHHHH sequences are additionally present at the N- and C-terminus, respectively.

### Protein expression and purification

All proteins were purified from the conditioned medium of episomally transfected 293-EBNA cells. Cells were cultured in Dulbecco's modified Eagle's medium supplemented with 10% fetal calf serum (Invitrogen), transfected using Fugene reagent (Roche Applied Science) and selected with 1 μg/ml of puromycin (Sigma). Proteins were purified by a combination of affinity and size-exclusion chromatography performed on an Äkta platform (GE Healthcare). Typically, 1.5 l of conditioned serum-free medium was loaded onto a 5-ml HisTrap column (GE Healthcare) equilibrated in phosphate-buffered saline (PBS) buffer, pH 7.45 (140 mM NaCl, 10 mM Na_2_PO_4_ and 3 mM KCl), and eluted with 500 mM imidazole in PBS. The eluate was concentrated using Vivaspin centrifugal devices (Sartorius AG) and further purified on a 24-ml Superdex 200 size-exclusion chromatography column (GE Healthcare) with Tris-buffered saline (TBS) buffer, pH 7.4, as the running buffer. Purified proteins were analysed by SDS-PAGE, quantified by measuring their absorption at 280 nm, concentrated to the final desired concentrations and flash-frozen in liquid nitrogen for storage at − 80 °C. Final yields were 10–20 mg of pure protein per litre of cell culture medium.

### Crystallisation and structure determination

NCAM ^1^FN3–^2^FN3 was concentrated to 13 mg/ml in TBS, and crystals were obtained by hanging drop vapour diffusion at room temperature using 2.2 M ammonium sulfate, 0.1 M sodium citrate, pH 5.2, 0.2 M potassium/sodium tartrate and 3–5% ethanol as precipitant. Crystals grew within 2 days and belong to space group *P*2_1_2_1_2_1_ with unit cell dimensions *a* = 52.77 Å, *b* = 71.35 Å and *c* = 98.22 Å. There are two ^1^FN3–^2^FN3 molecules in the asymmetric unit, resulting in a solvent content of ∼ 38%. Crystals were flash-frozen in liquid nitrogen after brief soaking in mother liquor supplemented with 20% glycerol. A crystal was soaked in mother liquor supplemented with 300 mM potassium iodide for 30 s before freezing to obtain a heavy atom derivative. Diffraction data from native and KI derivative crystals were collected at 100 K on station 14.1 at the Synchrotron Radiation Source (SRS) Daresbury and on station ID29 at the European Synchrotron Radiation Facility Grenoble, respectively. The NCAM ^1^FN3–^2^FN3 M610R mutant was concentrated to 14 mg/ml in TBS, and crystals were obtained by sitting drop vapour diffusion at room temperature using 2 M ammonium sulfate and 0.1 M sodium acetate, pH 4.6, as precipitant. Crystals grew within 3–4 days and belong to space group *P*2_1_2_1_2_1_ with unit cell dimensions *a* = 92.74 Å, *b* = 107.49 Å and *c* = 161.18 Å. There are six copies of mutant ^1^FN3–^2^FN3 in the asymmetric unit, resulting in a solvent content of ∼ 42%. Crystals were flash-frozen in liquid nitrogen after brief soaking in mother liquor supplemented with 20% glycerol, and diffraction data were collected at 100 K on station 10.1 at the SRS Daresbury. The diffraction data were processed with MOSFLM‡ and programs of the CCP4 suite.^48^ The structure of NCAM ^1^FN3–^2^FN3 was solved by single-wavelength anomalous dispersion phasing of a KI-soaked crystal using SHARP (Globalphasing Ltd., Cambridge) in full automatic mode. The structure was rebuilt with O^49^ and refined with Crystallography & NMR System.^50^ The structure of the NCAM M610R mutant was solved with some difficulty by molecular replacement with PHASER,^51,52^ using the isolated FN3 domains of the NCAM ^1^FN3–^2^FN3 structure as search models. Data collection, phasing and refinement statistics are summarised in Table 1. The figures were made with PyMOL§.

### SPR experiments

Binding experiments were performed on a Biacore 3000 instrument (GE Healthcare) at 25 °C. Proteins were immobilised on activated CM4 or CM5 chips using standard amine coupling procedures following the manufacturer's instructions. Briefly, flow cells were activated with 20 μl of a mixture of 0.2 M 1-ethyl-3-(3-dimethylaminopropyl)carbodiimide and 0.05 M *N*-hydroxy-sulfosuccinimide at a flow rate of 5 μl/min. The proteins to be immobilised (50–100 μg/ml in 10 mM sodium acetate, pH 4.5–5.5) were allowed to pass over activated flow cells to reach ∼ 2000–8000 RU, after which unreacted groups were blocked with 20 μl of 1 M ethanolamine, pH 8.5. Reference flow cells without protein were treated identically. The chips were equilibrated in 10 mM Hepes, pH 7.4, 150 mM NaCl, 50 μM ethylenediaminetetraacetic acid and 0.005% surfactant P20 (HBS-EP buffer), and serial dilutions of analyte proteins in PBS (our recombinant proteins or FGF1 from PeproTech) were injected at 20 μl/min for 300 s, followed by 200 s of pure buffer to monitor dissociation. Chips were regenerated using HBS-EP with 1 M glycine–HCl, pH 3.5. The sensorgrams were analysed with the BiaEvaluation 4.1 software.

### Protein Data Bank accession codes

Coordinates and structure factors for wild-type and M610R NCAM ^1^FN3–^2^FN3 have been deposited in the Protein Data Bank with codes 2vkw and 2vkx, respectively.

## Figures and Tables

**Table 1:** Crystallographic statistics

	Wild type	KI-soaked wild type	M610R mutant
Space group	*P*2_1_2_1_2_1_	*P*2_1_2_1_2_1_	*P*2_1_2_1_2_1_
Unit cell dimensions (Å)	*a* = 52.77	*a* = 51.43	*a* = 92.42
	*b* = 71.35	*b* = 73.00	*b* = 107.57
	*c* = 98.21	*c* = 97.31	*c* = 161.12
Beamline	SRS 14.1	European Synchrotron Radiation Facility ID29	SRS 10.1
Wavelength (Å)	1.49	1.50	1.12
Resolution (Å)	20–2.3 (2.42–2.30)^a^	20–2.1 (2.21–2.10)	20–2.7 (2.85–2.70)
Unique reflections	16,957	22,059	44,403
Multiplicity	4.8 (4.8)	14.8 (15.0)	6.2 (5.6)
Completeness (%)	99.5 (100)	99.9 (100)	99.0 (97.2)
*R*_merge_	0.057 (0.118)	0.098 (0.342)	0.082 (0.448)
Average *I*/σ(*I*)	19.7 (11.1)	25.1 (7.9)	17.9 (4.3)
Heavy atom sites		20	
Anomalous phasing power		1.6	
Figure of merit		0.40	
Protein atoms	3,058		9,264
Solvent sites	4 SO_4_^2−^, 98 H_2_O		11 SO_4_^2−^, 32 H_2_O
*R*_cryst_/*R*_free_	0.218/0.272		0.223/0.268
Average *B*-factor of protein atoms (Å^2^)	22.8		40.8
Average *B*-factor of solvent atoms (Å^2^)	27.2		57.5
r.m.s.d. bonds (Å)	0.006		0.007
r.m.s.d. angles (°)	1.4		1.4
r.m.s. difference *B*-factors (Å^2^)^b^	1.9		1.7
Ramachandran plot (%)^c^	89.8/10.2/0/0		88.3/11.1/0.4/0.2

a Values in parentheses refer to the highest-resolution shell.

b r.m.s. difference between *B*-factors of atoms connected by a covalent bond.

c Percentage of residues in most favoured, additionally allowed, generously allowed and disallowed regions.^32^

## Footnotes

† www.cbs.dtu.dk/services/NetOGlyc

‡ www.mrc-lmb.cam.ac.uk/harry/mosflm

§ www.pymol.org
